# Elevated basal AMP-activated protein kinase activity sensitizes colorectal cancer cells to growth inhibition by metformin

**DOI:** 10.1098/rsob.230021

**Published:** 2023-04-12

**Authors:** Kaitlin R. Morrison, Tingting Wang, Kuan Yoow Chan, Eleanor W. Trotter, Ari Gillespie, Michael Z. Michael, Jonathan S. Oakhill, Iain M. Hagan, Janni Petersen

**Affiliations:** ^1^ Flinders Health and Medical Research Institute, Flinders Centre for Innovation in Cancer, Flinders University, Adelaide, SA 5042, Australia; ^2^ Cancer Research UK Manchester Institute, Alderley Park, Macclesfield SK10 4TG, UK; ^3^ Flinders Centre for Innovation in Cancer, Dept. Gastroenterology and Hepatology, Flinders Medical Centre, Bedford Park, SA 5042, Australia; ^4^ Metabolic Signalling Laboratory, St Vincent's Institute of Medical Research, School of Medicine, University of Melbourne, Victoria 3065, Australia; ^5^ Mary MacKillop Institute for Health Research, Australian Catholic University, Victoria 3000, Australia; ^6^ Nutrition and Metabolism, South Australia Health and Medical Research Institute, Adelaide, SA 5000, Australia

**Keywords:** metformin, AMPK, *PRKAA1*, mTORC1, *α*1.S347A, colorectal cancers‌

## Abstract

Expression and activity of the AMP-activated protein kinase (AMPK) *α*1 catalytic subunit of the heterotrimeric kinase significantly correlates with poor outcome for colorectal cancer patients. Hence there is considerable interest in uncovering signalling vulnerabilities arising from this oncogenic elevation of AMPK*α*1 signalling. We have therefore attenuated mammalian target of rapamycin (mTOR) control of AMPK*α*1 to generate a mutant colorectal cancer in which AMPK*α*1 signalling is elevated because AMPK*α*1 serine 347 cannot be phosphorylated by mTORC1. The elevated AMPK*α*1 signalling in this HCT116 *α*1.S347A cell line confers hypersensitivity to growth inhibition by metformin. Complementary chemical approaches confirmed this relationship in both HCT116 and the genetically distinct HT29 colorectal cells, as AMPK activators imposed vulnerability to growth inhibition by metformin in both lines. Growth inhibition by metformin was abolished when AMPK*α*1 kinase was deleted. We conclude that elevated AMPK*α*1 activity modifies the signalling architecture in such a way that metformin treatment compromises cell proliferation. Not only does this mutant HCT116 *AMPKα1-S347A* line offer an invaluable resource for future studies, but our findings suggest that a robust biomarker for chronic AMPK*α*1 activation for patient stratification could herald a place for the well-tolerated drug metformin in colorectal cancer therapy.

## Graphical abstract

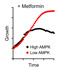

## 1. Introduction

Signalling from two main nutrient-sensing pathways in cells, the AMP-activated protein kinase (AMPK) and the mammalian target of rapamycin (mTOR) signalling pathways, is tightly coordinated to control cell growth and proliferation [1]. AMPK is a heterotrimeric kinase comprised *α*, *β* and *γ* subunits. Mammalian cells express several isoforms of all three subunits, of which *α*1 and *α*2 subunits contain the catalytic domain. AMPK is activated when intracellular adenosine monophosphate (AMP)/adenosine triphosphate (ATP) and adenosine diphosphate (ADP)/ATP ratios are elevated, e.g. when cells are nutritionally stressed [2]. The activation of AMPK blocks energy-consuming anabolic pathways, in part though inhibition of mTOR. AMPK signalling also promotes catabolic pathways such as autophagy, which recycle nutrients and re-establish cell homoeostasis [1]. mTOR is the catalytic subunit, of two functionally distinct multi-protein complexes. Each complex is defined by unique regulatory binding partners of which Raptor defines mTOR complex 1 (mTORC1) and Rictor defines mTOR complex 2 (mTORC2). Under nutrient replete conditions, mTORC1 drives anabolic (ATP-consuming) processes to support cell growth and proliferation [3]. We recently demonstrated that TORC1 directly phosphorylates an evolutionarily conserved residue serine (S367) in the fission yeast AMPK catalytic *α* subunit, Ssp2, to inhibit AMPK activity. We showed how this regulation is conserved in mammalian cells, as mTORC1 phosphorylates the equivalent mammalian AMPK *α*1-S347 and *α*2-S345 sites to reduce AMPK activity [4]. Thus, tight coordination of AMPK and mTORC1 activity in cells is in part controlled through feedback loops between these two nutrient-sensing pathways.

Worldwide, colorectal cancer is the third most prevalent cancer type. Patient survival when detected early is approximately 95%, however for patients with late-stage colorectal cancer, survival beyond 5 years is around 13%. For patients with intermediate stages II and III cancer, the 5-year survival rate is variable between 50 and 87% [5].

AMPK*α*1 is universally expressed in human cells, whereas AMPK*α*2 expression is highest in muscle tissue [6]. Hence, AMPK*α*1 is the main *α* isoform in cells of the human intestine. A recent study of colorectal cancer reported higher expression and activity of AMPK*α*1 in patients’ samples compared to their non-tumorous matched control samples [7]. They found that elevated AMPK*α*1 activity assists the survival of colorectal cancer cells under metabolic stress and high AMPK*α*1 activity significantly correlated with poor patient survival. Elevated AMPK*α*1 has also been observed in cervical and pancreatic cancers [8,9].

Metformin is well tolerated and the most common oral medication used to treat type 2 diabetes mellitus, as it reduces blood glucose levels through its effects on insulin and metabolic processes among others [10,11]. The use of metformin has been linked to decreased incidence of cancer in both diabetic and non-diabetic cohorts. Meta-analyses suggest that metformin decreases incidence of colorectal cancer [12,13], and metformin has also been linked to increased survival for pancreatic [14,15] and endometrial [16,17] cancer patients.

Metformin serum levels in diabetic recipients have been reported to reach 40 µM [18]. However, metformin accumulates in the lining of the human intestine at much higher concentrations. The concentration of metformin extracted from washed intestinal biopsies (ruling out external association of the drug with the tissue) from diabetic patients was up to 300 times greater than plasma concentration [19,20]. This accumulation of metformin in the intestinal lining enhances glucose uptake to confound PET imaging in patients taking metformin [21], because PET imaging relies upon glucose uptake. Hence, the effect of metformin in the intestine may differ to the rest of the body.

At the molecular level, metformin, in part, inhibits the mitochondrial electron transport chain complex 1, to prevent the production of ATP from AMP and ADP [22], causing AMP/ATP and ADP/ATP ratios to increase leading, in part, to AMPK activation. In addition, a range of AMPK-independent metformin effects have been reported including miRNA modulation, reduced reactive oxygen species (ROS) generation, suppression of angiogenesis, independent modulation mTORC1 activity, regulation of inflammation and glucose uptake [23,24].

The observations that high AMPK*α*1 activity significantly correlated with poor patient survival [7], and that metformin use in diabetic patients (which in part activates AMPK) reduced the incidence of colorectal cancer [12,13] merit greater scrutiny. We therefore wanted to characterize the relationship between AMPK*α*1 activity and metformin response in colon cancer cells, in further detail.

Here we show that CRISPR-edited AMPK *α*1-S347A phospho-blocking mutants that abolish the ability of mTORC1 to inhibit AMPK activity, display elevated basal AMPK activity in HCT116 cells. While *α*1-S347A cells are hypersensitive to metformin, specific AMPK activators A769662 and compound 991 have no impact on *α*1-S347A proliferation compared to wild-type HCT116 cells. Crucially, non-edited HCT116 and HT29 (colorectal cancer cell lines with different aetiology and mutational status) also gain sensitivity to metformin upon the elevation of basal AMPK activity. Hence, elevated basal AMPK activity in colorectal cancer cell alters the signalling landscape in such a way that metformin treatment compromises cell proliferation. Together, our data suggest that further research into the use of metformin for colorectal cancer patients with elevated AMPK activity may improve treatment outcomes.

## 2. Results

### 2.1. Blocking *α*1-S347 phosphorylation activates AMP-activated protein kinase in HCT116 cells

Metformin use in diabetics can protect against colorectal cancer [12,13]. However, metformin is a well-established activator of AMPK but elevated AMPK *α*1 activity is associated with poor patient outcome [7]. To further characterize the impact of metformin in cells with elevated AMPK *α*1 activity, we developed cell models of colorectal cancer with enhanced AMPK activity. We recently established that mTORC1 phosphorylates AMPK *α*1-S347 and that blocking this phosphorylation activates AMPK [4]. Using HCT116 colorectal cancer cells and CRISPR-Cas9 technology, we performed gene editing of the *α*1 genomic locus, the main *α* isoform in cells of the human intestine [6].

Our CRISPR method combined two published strategies, developed to improve selection and accuracy. This involved co-editing of the *ATP1A1* gene locus to generate *ATPA1-Q118RN129D* gain of function (GoF) mutations that render cells resistant to the glycoside ouabain. Thus, ouabain selection can be used to enrich for recombination competent cells that are likely to incorporate custom genetic modifications at an unlinked locus of interest [25]. Importantly, *ATPA1-Q118RN129D* mutations block the binding of ouabain but do not affect function of the Na^+^/K^+^ pump. Along with ouabain selection, we also used an eSpCas9-hGeminin fusion protein, where the C-terminus of the eSpCas9 enzyme was fused to amino acids 1–110 of human Geminin. The hGeminin fusion protein promotes eSpCas9 degradation in G1 of the cell cycle to prevent the formation of double-stranded breaks (DSBs) in G1, which would result in indel formation due to repair by non-homologous end joining. Thus, Cas9 is only present during the homology directed repair-competent phases of the cell cycle (S/G2/M) [26].

To validate the suitability of ouabain selection in our CRISPR screens, we first isolated independent *ATPA1-Q118RN129D* edited HCT116 cell lines and assessed AMPK activity by western blotting. Phosphorylation of T172 of the AMPK kinase domain and of S79 on ACC (AMPK substrate) was unaffected by the ATP1A1 GoF mutants (electronic supplementary material, figure S1*a*). The response to rapamycin inhibiting mTORC1 activity and therefore S6K-S389 phosphorylation (direct substrate of mTORC1) was also unaffected by the ATP1A1 mutations (electronic supplementary material, figure S1*b*). Furthermore, the growth rates of the three mutant cell lines were indistinguishable from wild-type (electronic supplementary material, figure S1*c*) as measured by live-cell imaging using the Incucyte® system. Therefore, the presence of the *ATPA1-Q118RN129D* mutations in our cell lines will not affect our interpretations of AMPK-dependent signalling; this is also supported by reports that the Na^+^/K^+^ pump remains functional with Q118R/N129D mutations [25,27].

To generate phospho-blocking alanine *AMPKα1-S347A*, phospho-mimetic glutamic acid *AMPKα1-S347E* mutations and the *AMPKα1* knockout (KO), we co-transfected with a plasmid harbouring the two guide RNAs and relevant repair template plasmids (see material and methods) to simultaneously mutate *ATP1A1* and *AMPKα1*. Screening of ouabain resistant clones by sequencing of genomic DNA identified cell lines harbouring all three of the desired mutations (electronic supplementary material, figure S2*a–d*). Western blotting with an antibody to the carboxy terminal region of AMPK*α*1 further confirmed the loss of AMPK *α*1 protein in the deletion strain (electronic supplementary material, figure S2*e*).

**Figure 1.**
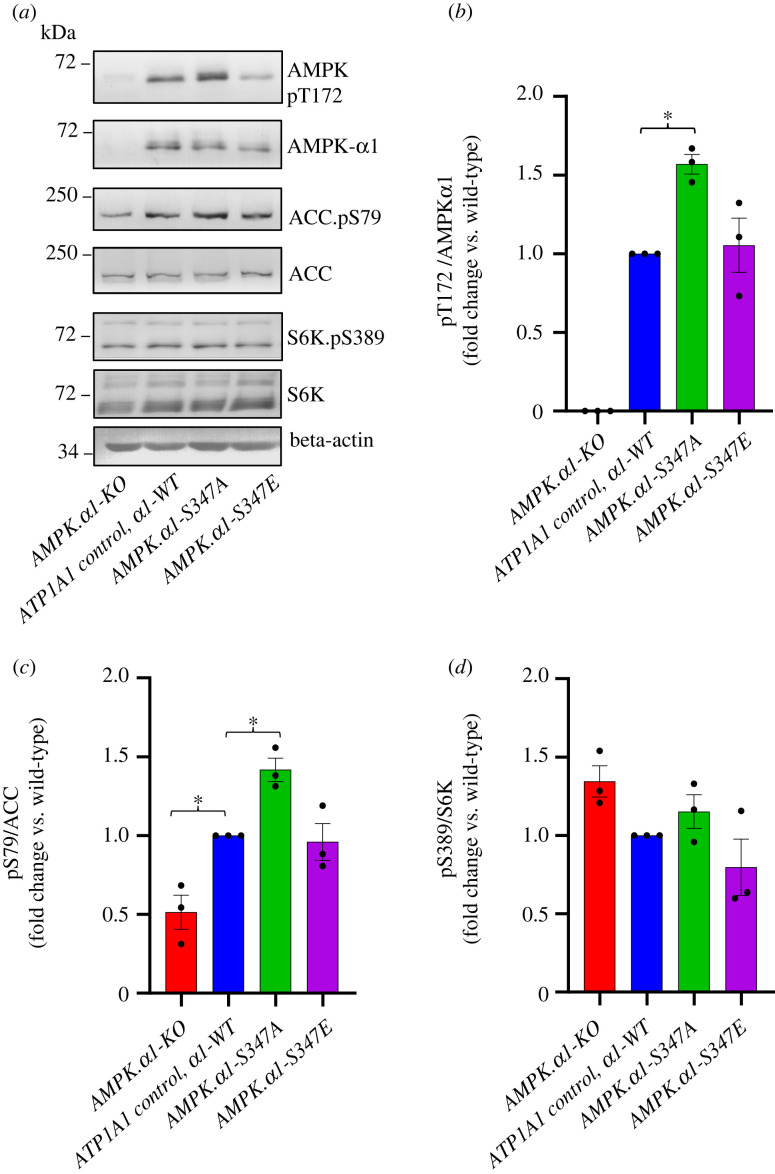
Blocking the inhibitory mTORC1 phosphorylation on *α*1-S347 enhances basal AMPK signalling in *AMPKα1-S347A* mutant cells. (*a*) Steady state protein lysates from CRISPR-edited HCT116 lines were extracted at 70–80% confluence and immunoblotted as indicated. Representative immunoblots are shown. Blots of AMPK, ACC and beta-actin are from the same SDS-PAGE gel, whereas phospho-specific blots are from the same protein extract loaded on separate gels. (*b–d*) Data are shown as mean fold change versus HCT116 ATP1A1 control ± s.e.m., *n = 3*. Statistical significance was calculated by Student's *t*-test versus HCT116 ATP1A1 control; *p < 0.05; only significantly different groups are shown.

Basal levels of AMPK signalling were assessed by phosphorylation on T172 in the kinase domain and by phosphorylation of the AMPK substrate ACC-S79. In *α*1-deletion cells, pT172 and pACC-S79 were dramatically reduced (figure 1*a–c*) consistent with *α*1 being the main isoform in colorectal cells. By contrast, pT172 and pACC-S79 were significantly augmented in *AMPKα1-S347A* mutant cells compared to the control ATP1A1 (GoF) AMPK*α*1 wild-type but were unchanged in *AMPKα1-S347E* mutant cells (figure 1*a–c*). Phosphorylation of S6K-S389 was unaffected in *AMPKα1-S347A* and *AMPKα1-S347E* mutant cells, and *AMPK1α1-KO* cells (figure 1*a*,*d*). In summary, the removal of the main AMPK *α* isoform reduces AMPK activity and increases basal mTORC1 signalling. By contrast, blocking the inhibitory mTORC1 phosphorylation on *AMPKα1-S347* enhances basal AMPK signalling in *AMPKα1-S347A* mutant cells confirming our previous conclusion [4]. Interestingly, the *AMPKα1-S347A* mutant does not enhance AMPK activity in mouse embryonic fibroblast [28], suggesting that the role of AMPK*α*1 serine 347 phosphorylation is context specific.

### 2.2. Elevated basal AMP-activated protein kinase signalling in *AMPK1α1-S347A* background sensitizes cells to metformin

**Figure 2.**
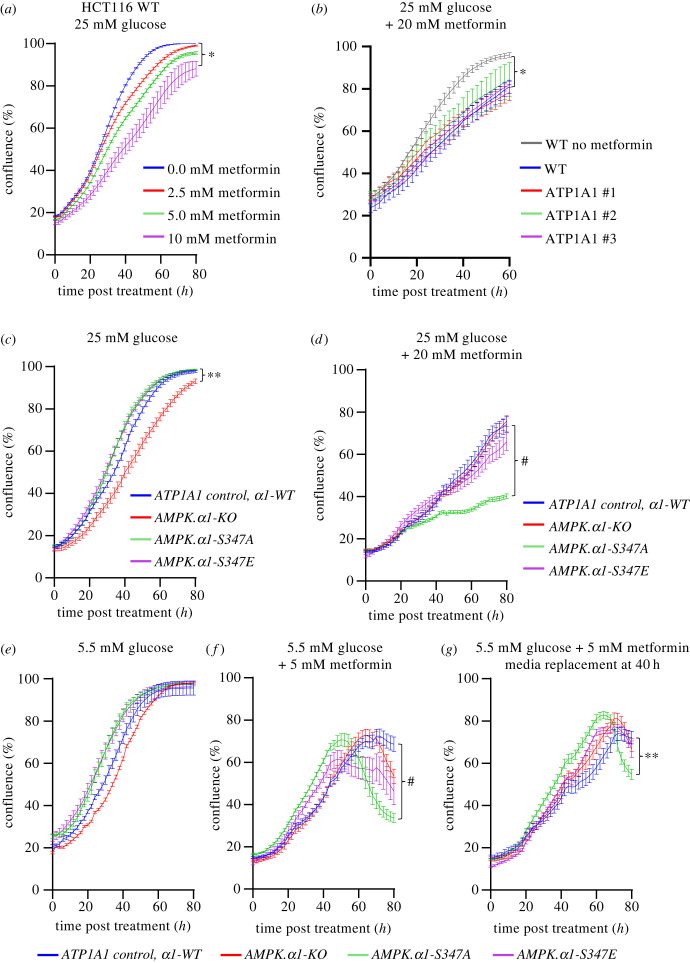
*AMPKα1-S347A* cells with elevated basal AMPK activity are hypersensitive to metformin. (*a–d*) Real-time proliferation analysis of indicated cell lines grown in 25 mM glucose DMEM with or without various concentrations of metformin. Data shown as mean confluence ± s.e.m. Statistical significance was calculated at endpoint (60 or 80 h) by two-way ANOVA versus 0.0 mM metformin or HCT116 ATP1A1 control with Dunnett's multiple-comparisons test; *p < 0.05. (*a*) Increased concentrations of metformin were added to WT HCT116 (*n* ≥ *6*). Statistical significance was shown for 0 mM versus 10 mM metformin. (*b*) Statistical significance was shown for WT HCT116 0 mM versus 20 mM metformin (*n* ≥ *3*). (*c*) CRISPR-edited HCT116 cells. Statistical significance shown for HCT116 ATP1A1 control versus AMPK *α*1-KO; **p < 0.01 (*n* ≥ *9*). (*d*) CRISPR-edited HCT116 cell lines with 20 mM metformin added. Statistical significance shown for HCT116 ATP1A1 control versus *AMPKα1-S347A;* ^#^*p* < 0.0001 (*n* ≥ *9*). (*e–g*) Real-time proliferation analysis of CRISPR-edited HCT116 cells in 5.5 mM glucose DMEM. Data are shown as mean confluence ± s.e.m. Statistical significance was calculated at endpoint (80 h) by two-way ANOVA versus HCT116 ATP1A1 control with Dunnett's multiple-comparisons test. (*e*) (*n* ≥ *6*). (*f*) CRISPR-edited HCT116 cells grown with 5 mM metformin. Statistical significance shown for HCT116 ATP1A1 control versus *AMPKα1-S347A* ^#^*p* < 0.0001 (*n* ≥ *7*). (*g*) CRISPR-edited HCT116 cells in 5.5 mM glucose DMEM + 40 h replacement. Statistical significance shown for HCT116 ATP1A1 control versus *AMPKα1-S347A;* **p < 0.01 (*n* ≥ *6*).

Metformin accumulates in the tissue lining of the human intestine at up to 300 times greater concentration than plasma concentration [19,20]. Hence, the impact of metformin in the intestine may differ from its impact in the rest of the body. Metformin reduces proliferation rates of HCT116 colorectal cells [29] seen here by the real-time analysis of proliferation using live-cell imaging in the Incucyte® system (figure 2*a*). Within 40 h, the growth/confluency of untreated wild-type HCT116 increased from 20% to 80% (fourfold) in contrast, increasing levels of metformin gradually reduced the gain in confluence (figure 2*a*). Upon the addition of 20 mM metformin (highest concentration used here) confluency increase from 25% to 60% (2.4-fold) after 40 h treatment, whereas untreated HCT116 cells increased from 25% to 90% (3.6-fold) in the same time frame (figure 2*b*). Proliferation rates for ATP1A1 (GoF) *α*1-WT (referred to as *control cells* from here on) were indistinguishable from wild-type HCT116 in the presence of 20 mM metformin (figure 2*b*). Notably, the impact of metformin seen here is consistent with previous reports of a 30% reduction in cell proliferation for HCT116 cells grown in 20 mM glucose to which 10 mM metformin was added [29].

The growth rates of *AMPKα1-S347A*, *AMPKα1-S347E* and control cells grown in standard DMEM 25 mM glucose were identical in untreated cells (figure 2*c*); however, proliferation of *AMPKα1-KO* cells was reduced. A recent report demonstrated a paradoxical activation of AMPK in colorectal cancer cells by 25 mM glucose that drives cell proliferation [30]; this was driven by the accumulation of ROS. Hence, the lack of AMPK*α*1 activation probably explains the reduced proliferation of the *AMPKα1-KO* cells when grown in 25 mM glucose (figure 2*c*).

Interestingly, when grown in 25 mM glucose the addition of 20 mM metformin to the *AMPKα1-S347A* mutant with high-basal levels of AMPK activity (figure 1*a,b*) radically reduced proliferation rates further compared to control cells (figure 2*d*). Therefore, high-basal AMPK activity appears to promote hypersensitivity to metformin.

Metformin's effect on cell proliferation is dependent on the level of glucose in the growth medium, with increased sensitivity to metformin seen at lower glucose levels [11,29]. We therefore grew cells in 5.5 mM glucose, to assess their sensitivity to metformin. All four cell lines; *AMPKα1-S347A, AMPKα1-S347E*, control cells and the *AMPKα1-KO* grew with similar growth rates in 5.5 mM glucose (figure 2*e*). The addition of 5 mM metformin promoted significantly faster cell death of the *AMPKα1-S347A* mutant after 50 h exposure to the drug (figure 2*f*), which again highlights the increased sensitivity of cells with high-basal AMPK activity to metformin. Two recent reports provided a molecular mechanism for metformin-induced cell death as they demonstrated that metformin induces cell death in lower glucose environments though PP2A, GSK3 and degradation of anti-apoptotic MCL-1 in HCT116 cells [29,31]**.**

Interestingly, increased glucose uptake is also part of the cellular response to metformin raising the possibility that cell death could be exaggerated because of glucose starvation. However, replacement with fresh identical media after 40 h, including 5.5 mM glucose and 5 mM metformin, did not significantly prevent cell death of the *AMPKα1-S347A* mutant HCT116 cell line (figure 2*g*).

In summary, the growth of the *AMPKα1-S347A* mutant cell line is sensitive to metformin in both high and low glucose. As intestinal metformin concentration is up to 300 times greater than plasma concentration, reported to reach 40 µM [18–20] metformin concentrations used here to demonstrate enhanced sensitivity of *α*1-S347A mutant cells are within the range of human therapeutic exposure in the gut.

### 2.3. Elevated basal AMP-activated protein kinase activity does not impact on sensitivity to specific AMP-activated protein kinase activators

**Figure 3.**
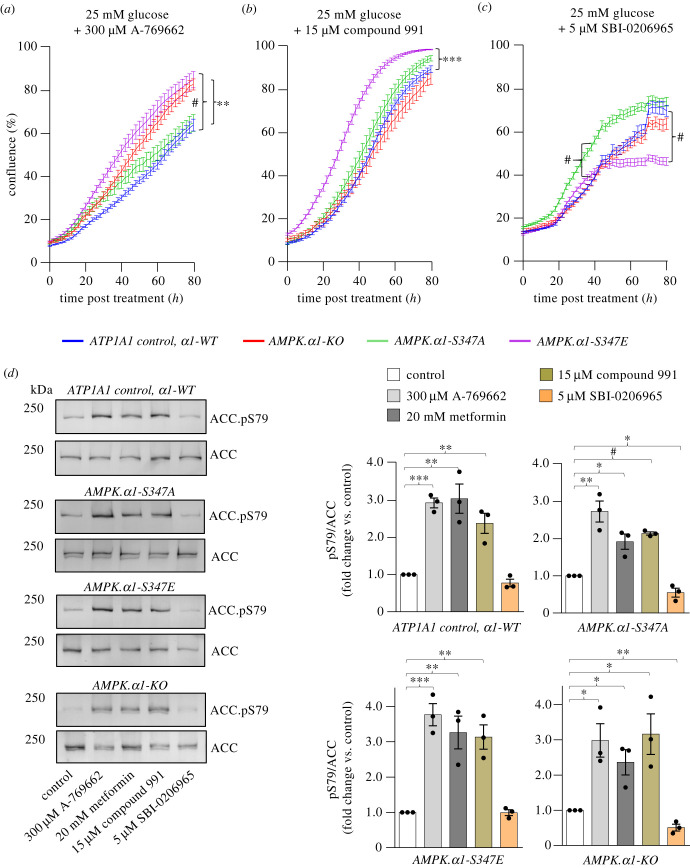
*AMPKα1-S347A* mutant cells are not responsive to further AMPK-specific activation. (*a–c*) Real-time proliferation analysis of CRISPR-edited HCT116 cells in 25 mM glucose DMEM. Data are shown as mean confluence ± s.e.m. Statistical significance was calculated by two-way ANOVA versus HCT116 ATP1A1 control with Dunnett's multiple-comparisons test. (*a*) 300 µM A-769662 was added. Statistical significance at endpoint (80 h) shown for HCT116 ATP1A1 control versus *AMPKα1-S347E* ^#^*p* < 0.0001 and for HCT116 ATP1A1 control versus *AMPKα1-KO*; **p < 0.01 (*n* ≥ *8*). (*b*) 15 µM Compound 991 was added. Statistical significance was shown at endpoint (80 h) for HCT116 ATP1A1 control versus *AMPKα1-S347E*; ***p < 0.001 (*n* ≥ *10*). (*c*) 5 µM SBI-0206965 was added. Statistical significance showed at endpoint (80 h) for HCT116 ATP1A1 control versus *AMPKα1-S347E*; ^#^*p* < 0.0001 and at 40 h for HCT116 ATP1A1 control versus *AMPKα1-S347A*; ^#^*p* < 0.0001 (*n* ≥ *12*). (*d*) Protein lysates from the CRISPR-edited HCT116 lines with indicated compounds added (6 h) were extracted at 70–80% confluence and immunoblotted as indicated. Representative immunoblots are shown. Data shown as mean fold change versus untreated control ± s.e.m., *n = 3*. Statistical significance was calculated by Student's *t*-test versus untreated control; *p < 0.05, **p < 0.01, ***p < 0.001, ^#^*p* < 0.0001; only significantly different groups are shown.

Metformin inhibits the mitochondrial electron transport chain complex 1, which elevates AMP and ADP levels to activate AMPK [22]. However, many AMPK-independent effects of metformin have been reported [11,23]. Therefore, the hypersensitivity of *AMPKα1-S347A* mutant cells to metformin could be because: (i) of elevated AMPK activity or (ii) metformin-regulated signalling (that is independent of AMPK) blocks proliferation in cells with high-basal AMPK activity. We therefore assessed the ability of two well-established specific AMPK activators A-769662 and C991 [32] to attenuate *AMPKα1-S347A* cell proliferation. The growth rate of *AMPKα1-S347A* was similar to control cells when 300 µM A-769662 was added (figure 3*a*). By contrast, A-769662 addition accelerated the growth rate of both *AMPKα1-S347E* and *AMPKα1-deletion* compared to wild-type *AMPKα1* controls. Enhanced growth of *AMPKα1-S347E* was also observed upon addition of 15 µM C991 (figure 3*b*). However, C991 also had no impact on cell proliferation in *AMPKα1-S347A* mutant cells relative to control cells (figure 3*b*). Next, we assessed the impact of both AMPK activators and metformin upon the amplitude of AMPK signalling by assessing ACC-Ser79 phosphorylation (direct substrate of AMPK [33]) following drug treatments. Metformin, A-7–769662 and C991 enhanced AMPK activity to similar levels in *AMPKα1-S347A*, *AMPKα1-S347E* and control cells (figure 3*d*). Hence enhanced growth of *AMPKα1-S347E* in response to specific AMPK activation is not due to altered acute enhancement of AMPK activity. Therefore, to uncover the reason why *AMPKα1-S347E* cells are resistant to AMPK activation further investigations will be required.

Metformin, A-769662 and C991, also enhanced the activity of the minor *α*2 isoform in AMPK *α*1-KO (figure 3*d*); however, importantly basal AMPK *α*2 activity is half that of control cells (figure 1*c*). Why *AMPKα1-KO* is only resistant to A-769662 and not C991 is not clear. A-769662 is generally accepted as *β*1 isoform specific [34] activator, maybe 991 is activating the remaining *α*2*β*2*γ*1 in *AMPKα1-KO*, whereas A-769662 cannot.

In summary, we do not observe enhanced AMPK activity in *AMPKα1-S347A* in response to metformin, hence AMPK activity cannot alone explain the hypersensitivity of *AMPKα1-S347A* to metformin (figure 2*d,f,g*).

Together these observations suggest that AMPK-independent signalling regulated by metformin combines with a specific signalling context that has been set by elevated basal AMPK activity in *AMPKα1-S347A* that blocks cell growth. This view is consistent with our observation of similar AMPK activities in *AMPKα1-S347A*, *AMPKα1-S347E* and control cells after all drug treatments (figure 3*d*) and the lack of impact on *AMPKα1-S347A* mutant cells proliferation in response to specific AMPK activators.

Next, we assessed the impact of AMPK inhibition on HCT116 cancer cell proliferation when basal AMPK activity is elevated. To this end, we used SBI-0206965. Although SBI-0206965 was originally identified as an inhibitor of the ULK1 kinase, it also inhibits AMPK activity [35,36]. Cells expressing the *AMPKα1-S347A* mutant grew faster, while *AMPKα1-S347E* mutant cells were sensitive to this compound, seen as significantly elevated or reduced cell proliferation, respectively, compared to control cells (figure 3*c*). Despite differences in growth rates, AMPK signalling assessed by ACC-S79 phosphorylation showed that AMPK activities were similar in *AMPKα1-S347A*, *AMPKα1-S347E* and control cells (figure 3*d*). This suggests that the level of basal AMPK activity may be key in setting the growth rate in response to both activation and inhibition of AMPK signalling.

### 2.4. Activation of AMP-activated protein kinase sensitizes non-edited HCT116 and HT29 colorectal cancer cells to metformin

**Figure 4.**
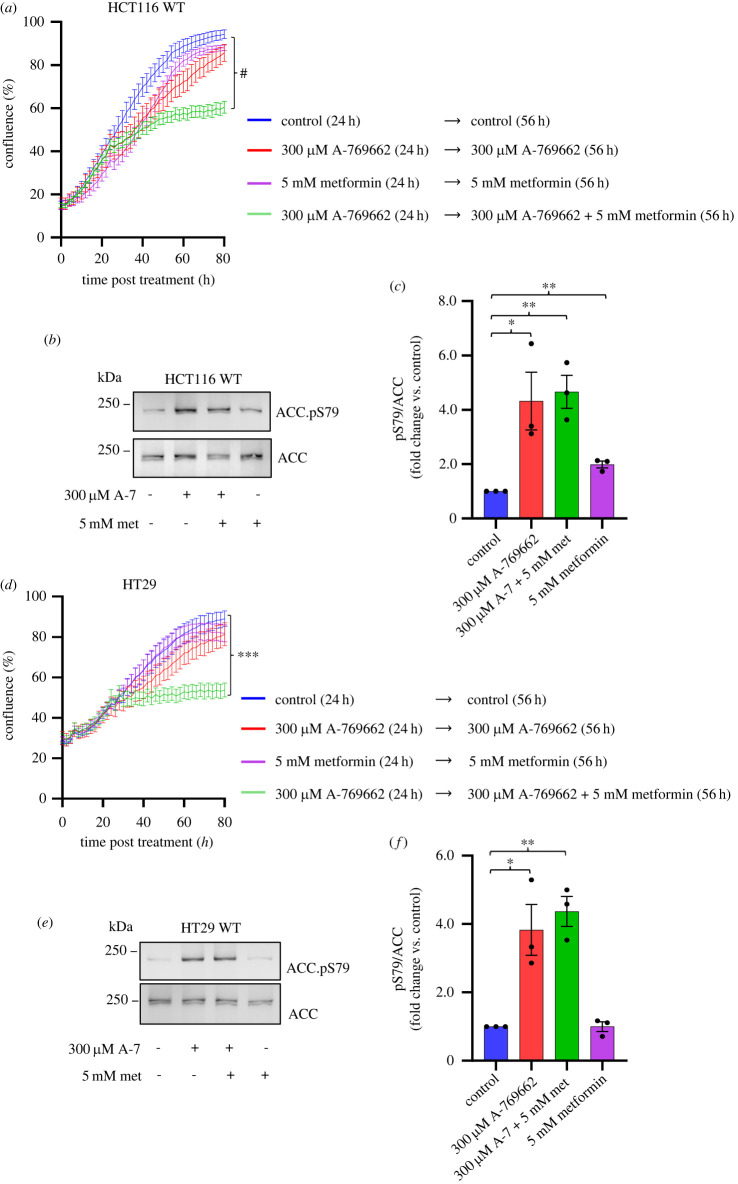
Activation of AMPK sensitises non-edited HCT116 and HT29 colorectal cancer cells to metformin. (*a*) Real-time proliferation analysis of wild-type HCT116 cells in 25 mM glucose DMEM with or without indicated compounds added. Cells were grown for 24 h with indicated treatments followed by replacement of culture media with or without indicated compounds. Data are shown as mean confluence ± s.e.m. Statistical significance was calculated by two-way ANOVA with Dunnett's multiple-comparisons test. Statistical significance at endpoint (80 h) shown for HCT116 non-treated control versus HCT116 with the activation of AMPK and metformin treatment; ^#^*p* < 0.0001 (*n* ≥ *5*). (*b,c*) Protein lysates from HCT116 cells with indicated compounds added (6 h) were extracted at 70–80% confluence and immunoblotted as indicated. Representative immunoblots are shown. Data are shown as mean fold change versus untreated control ± s.e.m., *n = 3*. Statistical significance was calculated by Student's *t*-test versus untreated control; *p < 0.05, **p < 0.01; only significantly different groups are shown. (*d*) Real-time proliferation analysis of wild-type HT29 cells in 25 mM glucose DMEM with or without indicated compounds added. Cells were grown for 24 h with indicated treatments followed by the replacement of culture media with or without indicated compounds. Data shown as mean confluence ± s.e.m. Statistical significance was calculated by two-way ANOVA with Dunnett's multiple-comparisons test. Statistical significance at endpoint (80 h) was shown for HT29 non-treated control versus HT29 with activation of AMPK and metformin treatment; ***p < 0. 001 (*n* ≥ *5*). (*e,f*) Protein lysates from HT29 cells with indicated compounds added (6 h) were extracted at 70–80% confluence and immunoblotted as indicated. Representative immunoblots are shown. Data are shown as mean fold change versus untreated control ± s.e.m.; *n = 3*. Statistical significance was calculated by Student's *t*-test versus untreated control; *p < 0.05, **p < 0.01, ^#^*p* < 0.0001; only significantly different groups are shown.

To further investigate whether elevated basal AMPK activity generates an intracellular signalling context that renders cells more sensitive to metformin, we treated two genetically diverse colorectal cancer cell lines with the specific AMPK activator A-769662 for 24 h before adding metformin. To this end, we used non-edited HCT116 cells that are oncogenic for *KRAS* but wild-type for *TP53* and HT29 cells that are wild-type for *KRAS* but mutant for *MYC* and *TP53* among many others. In both cell lines, 300 µM A-769662 and 5 mM metformin alone had limited impact on cell growth and confluence (figure 4*a,d*); however, when 5 mM metformin was with 300 µM A-769662, cell growth was reduced in both cell lines (figure 4*a,d*). The assessment of AMPK signalling by ACC pS79 demonstrated that AMPK activities were very similar and enhanced fourfold when both cell lines were exposed to 300 µM A-769662 only and when exposed to 300 µM A-769662 + 5 mM metformin (figure 4*b,c,e,f*).

Finally, treatment of HCT116 *AMPKα1* wild-type with 300 µM A-769662 but not of *AMPK α1-KO* sensitized cells to 5 mM metformin (electronic supplementary material, figure S3*a,b*). Together, these results support our previous conclusion that elevated basal AMPK signalling generates a signalling landscape that sensitizes cell proliferation to metformin.

### 2.5. Metformin-regulated signalling is predisposed to basal AMP-activated protein kinase activity

**Figure 5.**
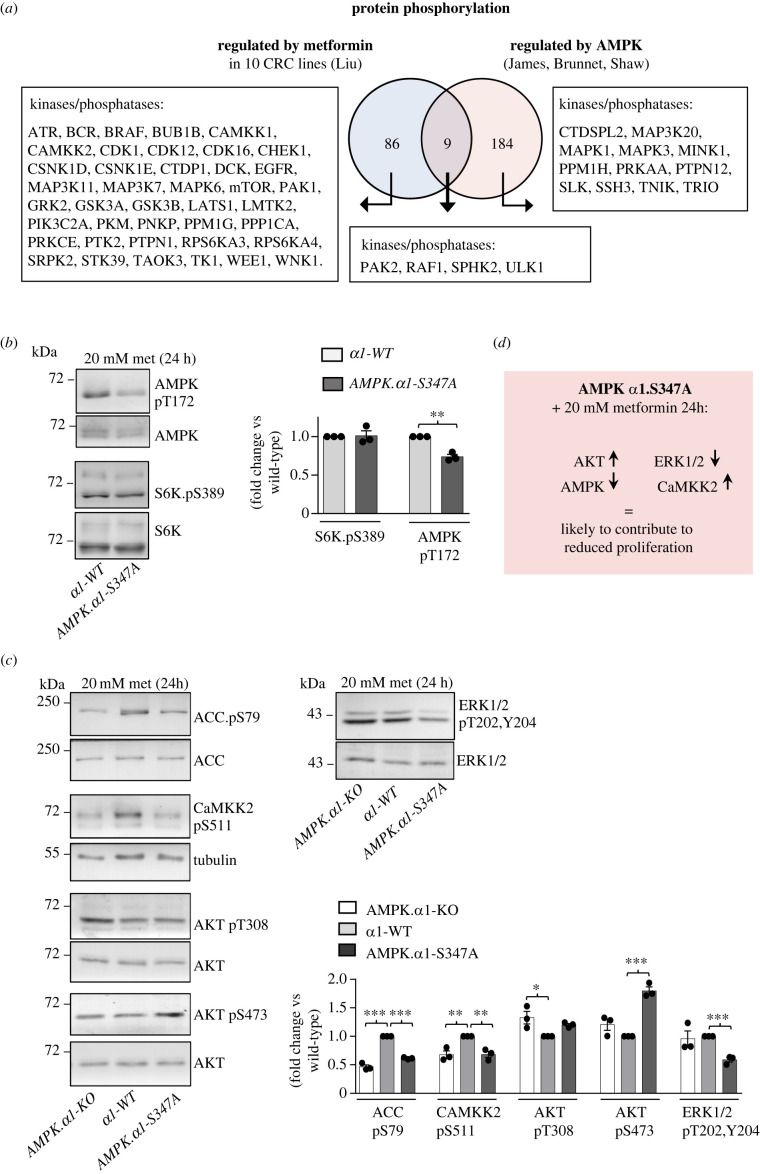
*AMPKα1-S347A* metformin-sensitive mutants have relatively low ERK signalling. (*a*) A larger number of signalling pathways are regulated by metformin and AMPK. A comparison of proteins reported to encompass AMPK and/or metformin (from 10 CRC cell lines) regulated phosphorylation [24,37–40]. Kinases and phosphatases are listed specifically. (*b,c*) Protein lysates from indicated cells grown with 20 mM metformin for 24 h were extracted at 70–80% confluence and immunoblotted as indicated. Representative immunoblots are shown. Data shown as mean fold change versus *AMPKα1-WT* ± s.e.m., *n = 3*. Statistical significance was calculated by Student's *t*-test versus *AMPKα1-WT*; *p < 0.05, **p < 0.01, ***p < 0.001; only significantly different groups are shown. (*d*) Compared to *AMPKα1-WT*, *AMPKα1-S347A* treated with metformin for 24 h has reduced ERK and AMPK but enhanced AKT and CaMKK2 activities, which may contribute to metformin hypersensitivity.

A very comprehensive study of metformin-regulated signalling in 10 different colorectal cancer cell lines has identified that the activity of more than 80 kinases and phosphatases form part of diverse signalling pathways that are regulated by metformin, across the 10 cell lines [24]. Direct changes to phosphorylation were demonstrated for more than 40 kinases and phosphatases in all cell lines (figure 5*a*, [24]). Similarly, several studies have investigated AMPK-dependent phosphorylation and over 180 AMPK-dependent substrates have been identified, including more than 15 kinases and phosphatases (figure 5*a*, [24,37–40]). Hence, both metformin and AMPK-dependent signalling are very complex.

The metformin-induced block to cell proliferation of cells with elevated AMPK activity begins to manifest after 24 h (figures 2*d* and 4*a,d*). With the aim of identifying the molecular mechanisms that contribute to metformin hypersensitivity of *AMPKα1-S347A* mutant cells, we assessed different aspects of cell signalling after 24 h treatment. To our surprise AMPK T172 phosphorylation was reduced in *AMPKα1-S347A* compared to wild-type after 24 h metformin treatment, while mTORC1 signalling was comparable to wild-type (figure 5*b*). Assessment of AMPK signalling by ACC pS79 established that AMPK activity in *AMPKα1-S347A* was reduced (figure 5*c*). However, *AMPKα1-KO* that are not sensitive to metformin (figure 2*d*) also have reduced AMPK activity (figure 5*c*) after 24 h of metformin. Hence the reduced AMPK activity cannot account for the sensitivity of *AMPKα1-S347A* mutant cells to metformin.

Phosphorylation of CaMKK2, an activator of AMPK [32], was previously shown to be regulated by metformin [24]; we therefore looked at CaMKK2 pS511 known to inhibit the kinase [41]; S511 phosphorylation was reduced in *AMPKα1-S347A* mutant cells (figure 5*c*) suggesting that CaMKK2 activity may be elevated in these cells. However, CaMKK2 pS511 phosphorylation was also significantly reduced in *AMPKα1-KO* cells thus cannot provide a sole explanation for *AMPKα1-S347A* sensitivity.

However, assessment of AKT activity based on mTORC2-regulated phosphorylation of S473 and PDK1 regulated T308 phosphorylation indicated that AKT activity is slightly elevated in *AMPKα1-S347A* cells (figure 5*c*). In addition, ERK1/2 signalling, which is known to promote cell proliferation was downregulated in *AMPKα1-S347A* mutant cells (figure 5*c*). Therefore, a combination of increased AKT and CaMKK2 and reduced ERK1/2 and AMPK activity, among many other possibilities (figure 5*a*), may contribute to metformin sensitivity of *AMPKα1-S347A* mutant cells (figure 5*d*).

## 3. Discussion

These data confirm our earlier findings [4] that blocking the ability of mTORC1 to phosphorylate serine 347 of *α*1 activates AMPK. Here, we demonstrate enhanced sensitivity to metformin in cells that harbour elevated basal AMPK activity. This impact is seen as reduced cell proliferation rates of *AMPKα1-S347A* mutant cells (figure 2*d*) and in two genetically diverse colorectal cancer cells lines HCT116 and HT29 when basal AMPK activities were elevated by chemical induction along with metformin exposure (figure 4*a,e*).

We demonstrate that cell proliferation of *AMPKα1-S347A* is only inhibited by metformin but not the specific AMPK activators A769662 and C991, suggesting that it is the combined high-basal AMPK activity and regulation of metformin specific targets which leads to reduced cell proliferation, rather than AMPK activation *per se*.

Metformin's effect on cell proliferation is dependent on the level of glucose in the growth medium, with increased sensitivity to metformin seen at lower glucose levels [29]. Interestingly, two recent papers have demonstrated that metformin induces apoptosis in colorectal cancer cell lines when glucose becomes limited [29,31]. A molecular mechanism for metformin-induced cell death was provided as they demonstrated that metformin induces cell death though PP2A, GSK3 and degradation of anti-apoptotic MCL-1 in HCT116 cells [29,31] Here, the addition metformin in low-glucose environments promoted significantly faster cell death of the *AMPKα1-S347A* mutant (figure 2*f*), again highlighting the increased sensitivity of cells with high-basal AMPK activity to metformin.

As expected, treatment with metformin elevated AMPK activity in all cell lines (figure 3*d*). The metformin-induced block to cell proliferation of cells with elevated basal AMPK activity starts to manifest after 24 h and so cannot be regarded as an acute block to cell proliferation (figures 2*d* and 4*a,e*). Interestingly, despite having higher basal AMPK activity (figure 1*a,c*), ACC-S79 phosphorylation in *AMPKα1-S347A* is reduced to *AMPKα1-KO* levels after 24 h metformin treatment suggesting that AMPK activity is reduced at the time metformin treatment starts to block growth in this cell line (figure 5*c*). However, this reduced AMPK activity in *AMPKα1-S347A* mutant cells cannot explain metformin sensitivity as growth of cells deleted of *α*1 is comparable to control cells (figure 2*d*). These observations support the notion that mechanisms stimulating metformin hypersensitivity are unrelated to metformin's ability to further activate AMPK. Instead, metformin hypersensitivity likely arises from a re-alignment of the signalling landscape by elevated basal AMPK *α*1 activity that promotes sensitivity to metformin.

Interestingly, ERK activity, that is known to promote proliferation, is reduced in *AMPKα1-S347A* mutant cells only (figure 5*c*; electronic supplementary material, figure S4*d*), supporting recent findings that enhanced AMPK activity reduces RAS signalling and therefore ERK1/2 activity [42]. This is regulated through AMPK-dependent degradation of HOXB9 that upregulates *KRAS* expression. Furthermore, global phospho-proteomic studies demonstrated that the phosphorylation of ERK2 (MAPK1) is regulated by AMPK signalling (figure 5*a*).

Previous studies have demonstrated how a large number of enzymes and signalling pathways are regulated by AMPK activity and metformin (figure 5*a*) [11,24,37–40]. Therefore, the specific combination of AMPK and metformin-regulated signalling pathways responsible for the reduced cell proliferation observed here are likely to arise from the combined impact at several nodes in these diverse signalling networks. Low ERK1/2 in combination with low AMPK, and elevated AKT and CaMKK2 (figure 5*d*) may contribute to reduced cell proliferation in *α*1-S347A mutant cells exposed to metformin.

The HCT116 cell line used in this study is driven in part by aberrant KRAS and WNT signalling [43,44] and exhibits microsatellite instability [45]; however, HT29 is wild-type for *KRAS* but mutant for *MYC* and *TP53*, AMPK activation in both cells lines sensitizes them to 5 mM metformin, a concentration that on its own had no major effect on cell proliferation in these conditions (figure 4*a,e*). Such independence of the impact of metformin from the nature of the transforming complexion of the cell line strongly suggests that biomarkers of elevated AMPK *α*1 activity alone will be sufficient to predict a positive outcome from metformin therapy.

Together, our data demonstrate that activation of basal AMPK signalling is sufficient to achieve metformin hypersensitivity. Interestingly, these findings are consistent with the meta-analyses suggesting metformin is linked to decreased incidence of colorectal cancer along with the finding that elevated AMPK*α*1 activity is seen frequently in colorectal adenocarcinomas [7]. Our data suggest that more research into whether colorectal cancer patients with enhanced tumoral AMPK activity may benefit from treatment with the well-tolerated metformin is warranted. It may be the case that for people treated with metformin, mutant cells of the intestine that contain elevated AMPK activity, (associated with poor prognosis [7]) may also be preferentially eliminated by metformin. Recent clinical trials treating colorectal cancer with metformin as a monotherapy have had diverse outcomes [11]; however, stratification of the patient cohort based upon elevated tumour AMPK *α*1activity may yet reveal a place for metformin as a mono or combination therapy for future trials.

## 4. Materials and methods

### 4.1. Design and preparation of CRISPR-Cas9 vectors, guide RNAs and ssODNs

The CRISPR-Cas9 plasmid used in this study was eSpCas9-hGeminin. The plasmid is a modified version of the eSpCas9(1.1)_No_FLAG_ATP1A1_G3_Dual_sgRNA plasmid (addgene plasmid #86613) (https://www.addgene.org/86613/), where the C-terminus of the eSpCas9 enzyme was fused to amino acids 1–110 of human Geminin. The hGeminin fusion protein promotes eSpCas9 degradation in G1 of the cell cycle. This prevents the formation of DSBs in G1 which would result in indel formation due to NHEJ. Thus, Cas9 is present during HDR-favoured phases of the cell cycle (S/G2/M) [26]. The eSpCas9-hGeminin plasmid expresses the *ATP1A1* G3 gRNA to allow for the targeted editing of the *ATP1A1* gene. An ATP1A1 template plasmid was used to generate naturally occurring mutations in the *ATP1A1* gene (encoding the sodium/potassium pump Na+/K + ATPase), conferring resistance to the drug ouabain (Sigma, #O3125) without changing its enzymatic function [25]

*PRKAA1* gRNA sequences were designed using the software from http://crispor.tefor.net/crispor.py, and 20 bp guides within 100 bp of the region of interest beginning with G (due to the use of a U6 promoter) were selected. 5′-CACC-3′ and 5′-AAAC-3′ were appended to the sense and antisense guide sequences, respectively. For cell lines with point mutations at amino acid Ser347 in *PRKAA1*, the following gRNAs were generated at exon 7 (NC_00005.10, exon7: 40 764 929–40 764 948); sense: 5′-CACCGAATGGTACTCTTTCAGGAT-3′ and antisense: 5′-AAACATCCTGAAAGAGTACCATTC-3′. For *PRKAA1* KO cell lines, the following gRNAs were generated at exon 2 (NC_00005.10, exon2: 40 777 568–40 777 587); sense: 5′-CACCGTTGGCAAACATGAATTGAC-3′ and antisense: 5′-AAACGTCAATTCATGTTTGCCAAC-3′. Annealed gRNAs were cloned into the eSpCas9-hGeminin plasmid and transformed in *Escherichia coli*, under ampicillin selection. Plasmids were harvested from transformed colonies using the QIAprep Spin Miniprep Kit (Qiagen, #27104) and correct insertion of the gRNA was assessed by Sanger sequencing at the Flinders Sequencing Facility using the primer 5′-TATGCTGAATTACAGAACTCGG-3′. Successful plasmids were amplified for harvesting by the CompactPrep Plasmid Midi Kit (Qiagen, #12843) for use in transfection. To generate point mutations in the *PKRAA1* gene to modify Ser347, 200 bp single-stranded oligonucleotide (ssODN) donors were purchased through Integrated DNA Technologies, Inc., which included silent mutations in the PAM, gRNA (to reduce re-editing) and restriction site (added for screening purposes) sequences.

### 4.2. CRISPR Cas-9 gene editing

HCT116 (European Collection of Authenticated Cell Cultures, #91091005) cells were maintained in DMEM (Sigma-Aldrich, #D6429) supplemented with 10% FBS (Life Technologies, #10099141) and 1 : 100 Penicillin Streptomycin (Sigma, #P4333) in an incubator at 37°C and 5% CO_2_. Cells were passaged twice weekly.

For transfection using CRISPR-Cas9 gene editing, cells were harvested by trypsinization at 70–90% confluence. 5 × 10^6^ cells were co-transfected with 10 µl plasmid DNA (eSpCas9-hGeminin – gRNA and ATP1A1 template at 1 µg µl^–1^), plus 5 µl ssODN donor (100 µM) for point mutation edits. Cells were electroporated using the Neon Transfection System (Invitrogen, #MPK5000) at 1400 V, 30 ms, 1 pulse. Electroporated cells were incubated in 15 cm round dishes for 48–72 h, then treated with ouabain selection media (10 µM) which was replaced every few days for 5–7 days. Colonies were picked under the microscope and placed in 48-well plates without selection media, then expanded to six-well plates for cryopreservation and DNA and/or protein analysis.

### 4.3. Live-cell imaging growth assays

For cell growth assays, HCT116 cells were seeded in 96-well plates at 6 × 10^3^ cells/well and their growth tracked by live-cell imaging in the Incucyte® system (Panasonic, #MCO-20AIC-PE). Cells were challenged with various nutrient and drug conditions at 10–20% confluence using the following reagents: DMEM (Sigma-Aldrich, #D6429), DMEM – low glucose (Sigma, #D5546), SBI-0206965 (Sigma, #SML1540), metformin hydrochloride (Glentham Life Sciences, #GP2747), A-769662 (Abcam, #ab120335) and Compound 991 (AOBIOUS, #AOB8150). All graphs shown are produced from greater than or equal to three biological repeats, wells with identical starting confluency were included.

### 4.4. Isolation of genomic DNA, PCR amplification and screening of CRISPR-Cas9-edited *PRKAA1* genes

Cell pellets were lysed in lysis buffer (20 mM Tris-HCl, pH 8.0, 1 mM EDTA, 0.67% (w/v) SDS, and 124 µg/ml proteinase K (Roche Life Science, #3115879) for 4 h at 55°C and precipitated with 2.5 x ice-cold 100% ethanol and 0.1 x NaOAc. For purer DNA, DNA was harvested using the Monarch Genomic DNA Purification Kit (New England BioLabs, #T3010). To screen point mutations at the *AMPK*-α1.S347A locus, a 621 bp region in *PRKAA1* exon 7 was amplified using the primers F: 5′-TGAATGGTTTAAACAGGACC-3′ and R: 5′-CGTACACGCAAATAATATGGG-3′. The region was amplified using Q5 polymerase (New England BioLabs, #M0491) using the following conditions: 98°C 30 s; 40 cycles of 98°C 30 s, 58.8°C 60 s and 72°C 40 s; and 72°C 5 min. The PCR product was digested with XbaI (New England BioLabs, #R0145) to confirm insertion of the ssODN. To validate the correct insertion of point mutations, samples were sequenced using the primer 5′-TGAATGGTTTAAACAGGACC-3′ by Sanger sequencing at the Flinders Sequencing Facility.

After screening by western blot, to confirm the genotype of AMPK-α1.KO mutants, a 1659 bp region in *PRKAA1* exon 2 was amplified using the primers F: 5′-CCTTCCCCTCTTCCTTTAGTCTTC-3′ and R: 5′-CATGTGGGCATTTGGAGG TC-3′. The region was amplified using Q5 polymerase at 98°C 30 s; 40 cycles of 98°C 10 s, 57.3°C 30 s and 72°C 60 s; and 72°C 2 min. Samples were sequenced using the primer 5′-TAGTAGAGATGGGGTTTTGC-3′ by Sanger sequencing. Human reference sequences were downloaded from NCBI Aceview.

### 4.5. Western blotting of mammalian cells total protein extracts

For cells grown for western blotting analysis, HCT116 cells were seeded in six-well plates and harvested at 70% confluence by using the trichloroacetic acid precipitation protocol [46]. Primary antibodies were incubated at 4°C overnight, and alkaline phosphatase- or horseradish peroxidase-conjugated secondary antibodies were incubated at RT for 1 h. Primary antibodies (see materials) were used at the following dilutions: rabbit anti-AMPK-alpha1 (1 : 1000), rabbit anti-phospho-AMPK-Thr172 (1 : 2000), rabbit anti-ACC (1 : 1000), rabbit anti-phospho-ACC-Ser79 (1 : 1000), rabbit anti-p70 S6K (1 : 2000), rabbit anti-phospho-p70 S6K-Thr389 (1 : 500) and mouse anti-β-actin (1 : 1000) rabbit anti-ERK1 (1 : 1000), rabbit anti-phospho-ERK-Thr202-Tyr240 (1 : 2000), rabbit anti-AKT (1 : 1000), rabbit anti-phospho-AKT-Thr308(1 : 1000), rabbit anti-phospho-AKT-Ser473(1 : 2000), rabbit anti-phospho-CaMKK2-Se511(1 : 500). Signal detection was achieved using NBT/BCIP and HRP substrates on PVDF membranes. For higher molecular weight protein i.e. ACC, 7.5% acrylamide gels were used.

### 4.6. Materials

**Table 1.** Materials.

resource	source	identifier
*antibodies*		
rabbit anti-AMPK-alpha1	Cell Signalling Technology Inc.	#2795S
rabbit anti-phospho-AMPK-Thr172	Millipore UK Ltd	#07681
rabbit anti-ACC	Cell Signalling Technology Inc.	#3637
rabbit anti-phospho-ACC-Ser79	Cell Signalling Technology Inc.	#3661
rabbit anti-p70 S6K	Cell Signalling Technology Inc.	#9202
rabbit anti-phospho-p70 S6K-Thr389	Cell Signalling Technology Inc.	#9205
mouse anti-β-actin	Abcam	#ab8224
mouse anti-ERK	Cell Signalling Technology Inc.	#46965
rabbit anti-phospho-ERK-Thr 202.Tyr204	Cell Signalling Technology Inc.	#9101S
rabbit anti-AKT	Cell Signalling Technology Inc.	#4691S
rabbit anti-phospho-AKT-Thr308	Cell Signalling Technology Inc.	#9275S
rabbit anti-phospho-AKT-Thr473	Cell Signalling Technology Inc.	#4058S
rabbit anti-phospho-CaMKK2-Ser511	Cell Signalling Technology Inc.	#12818S
goat anti-rabbit IgG-alkaline phosphatase	Sigma-Aldrich	#A3687
goat anti-mouse IgG-alkaline phosphatase	Sigma-Aldrich	#A3562
goat anti-rabbit IgG-peroxidase	Sigma-Aldrich	#A0545
*bacterial strains*		
*Escherichia coli* MAX Efficiency DH5*α* Competent Cells	ThermoFisher Scientific	#18258012
*commercial assays*		
QIAprep Spin Miniprep Kit	Qiagen	#27104
CompactPrep Plasmid Midi Kit	Qiagen	#12843
Monarch Genomic DNA Purification Kit	New England BioLabs	#T3010
Q5 High-Fidelity DNA Polymerase	New England BioLabs	#M0491
*experimental models*		
HCT116 cells	European Collection of Authenticated Cell Cultures	#91091005
*oligonucleotides*		
CRISPR plasmid sequencing primer (JP#1286): TATGCTGAATTACAGAACTCGG	Sigma-Aldrich	Addgene plasmid #86613
human AMPK*α*1 F (JP#1300): TGAATGGTTTAAACAGGACC	Sigma-Aldrich	*PRKAA1* NC_00005.10, exon7: 40 765 213–40 765 232
human AMPK*α*1 R (JP#1324): CGTACACGCAAATAATATGGG	Sigma-Aldrich	*PRKAA1* NC_00005.10, exon8: 40 764 612–40 764 632
human AMPK*α*1 sense guide (JP#1302): CACCGAATGGTACTCTTTCAGGAT	Sigma-Aldrich	*PRKAA1* NC_00005.10, exon7: 40 764 929–40 764 948
human AMPK*α*1 antisense guide (JP#1303): AAACATCCTGAAAGAGTACCATTC	Sigma-Aldrich	*PRKAA1* NC_00005.10, exon7: 40 764 929–40 764 948
human AMPK*α*1.S347A donor (JP#1314): CATCTCATAATAGATAACAGGAGAATAATGAATGAAGCCAAAGATTTCTATTTGGCGACAGCCCCACCTGATTCTTTTCTAGATGATCATCACCTGACTCGGCCTCATCCTGAAAGAGTTCCGTTTTTGGTTGCTGAAACACCAAGGGCACGCCATACCCTTGATGAATTAAATCCACAGAAATCCAAACACCAAGGTGT	Integrated DNA Technologies	*PRKAA1* NC_00005.10, exon7: 40 764 855–40 765 054
human AMPK*α*1.S347E donor (JP#1315): CATCTCATAATAGATAACAGGAGAATAATGAATGAAGCCAAAGATTTCTATTTGGCGACAGAACCACCTGATTCTTTTCTAGATGATCATCACCTGACTCGGCCTCATCCTGAAAGAGTTCCGTTTTTGGTTGCTGAAACACCAAGGGCACGCCATACCCTTGATGAATTAAATCCACAGAAATCCAAACACCAAGGTGT	Integrated DNA Technologies	*PRKAA1* NC_00005.10, exon7: 40 764 855–40 765 054
human AMPK*α*1-KO sense guide (JP#1340): CACCGTTGGCAAACATGAATTGAC	Sigma-Aldrich	*PRKAA1* NC_00005.10, exon2: 40 777 568–40 777 587
human AMPK*α*1-KO antisense guide (JP#1341): AAACGTCAATTCATGTTTGCCAAC	Sigma-Aldrich	*PRKAA1* NC_00005.10, exon2: 40 777 568–40 777 587
human AMPK*α*1-KO F (JP#1383): TAGTAGAGATGGGGTTTTGC	Sigma-Aldrich	*PRKAA1* NC_00005.10, intron1: 40 777 752–40 777 772
human AMPK*α*1-KO R (JP#1392): CATGTGGGCATTTGGAGGTC	Sigma-Aldrich	*PRKAA1* NC_00005.10, intron2: 40 776 809–40 776 828
human AMPK*α*1-KO F (JP#1393): CCTTCCCCTCTTCCTTTAGTCTTC	Sigma-Aldrich	*PRKAA1* NC_00005.10, intron1: 40 778 444–40 778 467
*Reagents*		
Ouabain octahydrate	Sigma-Aldrich	#O3125
ampicillin sodium salt	ThermoFisher Scientific	#08390010
DMEM	Sigma-Aldrich	#D6429
FBS	Life Technologies	#10099141
penicillin streptomycin	Sigma-Aldrich	#P4333
DMEM – low glucose	Sigma-Aldrich	#D5546
SBI-0206965	Sigma-Aldrich	#SML1540
Metformin hydrochloride	Glentham Life Sciences	#GP2747
A-769662	Abcam	#ab120335
Compound 991	AOBIOUS	#AOB8150
Proteinase K	Roche Life Science	#3115879
XbaI	New England BioLabs	#R0145
NBT	Chem Supply	#GC4263
BCIP	Chem Supply	#GC5463
SignalFire Elite ECL Reagent	Cell Signalling Technology	#12757
*Recombinant DNA*		
eSpCas9-hGeminin	Hagan Lab	https://www.addgene.org/199344/
ATP1A1 template plasmid	Hagan Lab	
*software*		
CRISPOR (v4.99)	Tefor Infrastructure	http://crispor.tefor.net/crispor.py
*other*		
Neon transfection system	Invitrogen	#MPK5000
Incucyte system	Panasonic	#MCO-20AIC-PE

## Data Availability

The data are provided in the electronic supplementary material [47].
